# Specific Substitutions in Region V2 of gp120 *env* confer SHIV Neutralisation Resistance

**DOI:** 10.3390/pathogens9030181

**Published:** 2020-03-03

**Authors:** Yalcin Pisil, Zafer Yazici, Hisatoshi Shida, Shuzo Matsushita, Tomoyuki Miura

**Affiliations:** 1 Laboratory of Primate Model, Research Center for Infectious Diseases, Institute for Frontier Life and Medical Science, Kyoto University, Kyoto 615-8530, Japan; yalcin.pisil@gmail.com; 2 Department of Virology, Faculty of Veterinary Medicine, 19 Mayis University, Samsun 55270, Turkey; zyazici@omu.edu.tr; 3 Division of Molecular Virology, Institute of Immunological Science, Hokkaido University, Hokkaido 060-0808, Japan; hmyy2010@yahoo.co.jp; 4 Center for AIDS Research, Kumamoto University, Kumamoto 860-8555, Japan; shuzo@kumamoto-u.ac.jp

**Keywords:** AIDS, SHIV, HIV, mutagenesis, CCR5, Env, V2 region, Rhesus macaque

## Abstract

A tier 2 SHIV-MK38 strain was obtained after two in vivo passages of tier 1 SHIV-MK1. SHIV-MK38#818, cloned from the MK38 strain, was neutralisation-resistant, like the parental MK38 strain, to SHIV-infected monkey plasma (MP), HIV-1-infected human pooled plasma (HPP), and KD247 monoclonal antibody (mAb) (anti-V3 gp120 *env*). We investigated the mechanisms underlying the resistance of #818, specifically the amino acid substitutions that confer resistance to MK1. We introduced amino acid substitutions in the MK1 envelope by in vitro mutagenesis and then compared the neutralisation resistance to MP, HPP, and KD247 mAb with #818 in a neutralisation assay using TZM-bl cells. We selected 11 substitutions in the V1, V2, C2, V4, C4, and V5 regions based on the alignment of *env* of MK1 and #818. The neutralisation resistance of the mutant MK1s with 7 of 11 substitutions in the V1, C2, C4, and V5 regions did not change significantly. These substitutions did not alter any negative charges or N-glycans. The substitutions N169D and K187E, which added negative charges, and S190N in the V2 region of gp120 and A389T in V4, which created sites for N-glycan, conferred high neutralisation resistance. The combinations N169D+K187E, N169D+S190N, and N169D+A389T resulted in MK1 neutralisation resistance close to that of #818. The combinations without 169D were neutralisation-sensitive. Therefore, N169D is the most important substitution for neutralisation resistance. This study demonstrated that although the V3 region sequences of #818 and MK1 are the same, V3 binding antibodies cannot neutralise #818 pseudovirus. Instead, mutations in the V2 and V4 regions inhibit the neutralisation of anti-V3 antibodies. We hypothesised that 169D and 190N altered the MK1 Env conformation so that the V3 region is buried. Therefore, the V2 region may block KD247 from binding to the tip of the V3 region.

## 1. Introduction

Simian immunodeficiency virus (SIV) has been used to study the pathogenesis of AIDS, and in vaccine development. However, the Env structures and antigenicity of SIV and HIV differ and have low homology. Therefore, this model is inappropriate for analysing the role of neutralising antibody in protective immunity. To overcome this problem, the HIV-1 *env* gene was inserted into the SIV genome, which lacks its own *env* gene, and simian/human immunodeficiency viruses (SHIVs) were constructed [1,2].

SHIVs were infectious in rhesus macaques (*Macaca mulatta*) and became highly pathogenic after in vivo adaptation [3,4]. Nevertheless, the initial SHIV89.6p macaque model could not predict the effects of candidate vaccines in the human clinical trial (STEP trial) conducted by Merck in 2007. This failure was mainly attributed to differences in coreceptor use and resistance to neutralising antibodies between SHIV89.6p and HIV-1. SHIV89.6p uses CXCR4 (X4) as the main coreceptor and is sensitive to neutralising antibodies (tier 1), whereas circulating HIV-1s use CCR5 (R5) and are antibody-resistant (tier 2) [5].

In 2010, Matsuda et al. constructed R5 tropic SHIV-MK1 via five amino acid substitutions in the *env* V3 region of tier 1 X4 tropic SHIV-KS661, based on the consensus amino acid alignment analyses of subtype B R5 HIV-1; however, MK1 was still tier 1 [6]. Therefore, they passaged MK1 in vivo and obtained a tier 2 MK38 strain as a mixture of viruses after two passages. Then, MK38#818, which is neutralisation-resistant to HIV-1-infected human plasma, similar to the parental SHIV-MK38, was cloned from the MK38 strain [5].

**Figure 1:**
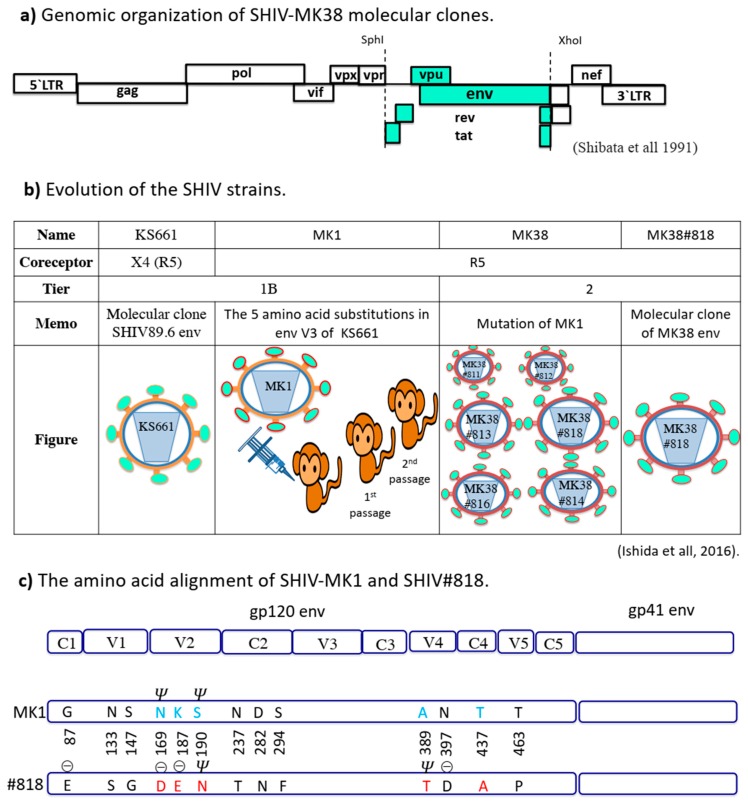
Summary of the SHIV research. (**a**) Genomic organisation of SHIV-MK38 molecular clones generated by replacing the *Sph*I/*Xho*I fragment of SHIV-MK38 (turquoise blue) with the SHIV-KS661 backbone (in white box). (**b**) Evolution of the SHIV strains. (**c**) Amino acid alignment of MK1 env and #818 env. G, glycine; N, asparagine; S, serine; K, lysine; D, aspartic acid; A, alanine; T, threonine; E, glutamic acid; F, phenylalanine; P, proline; ⊝, negative charge; *Ψ*, N glycan. Blue and red letters indicate the substitutions.

Matsuda et al. compared the gp120 V1, V2, and V3 amino acid alignments of SHIV-KS661 and 14 SHIV-MK38 clones. On checking the positions of the amino acid substitutions in the 14 clones, the majority of the MK38 clones contained substitutions in the V1 region, and some had substitutions in V2 [6]. Subsequently, Ishida et al. sequenced the complete *env* genes of the MK38 series and identified the substitutions between SHIV-MK1 and MK38#818 (Figure 1c). However, neither Ishida et al. nor Matsuda et al. examined the roles of the substitutions in neutralisation resistance.

Here, we investigated the cause of the resistance of #818, specifically the amino acid substitutions that make MK1 resistant. We introduced amino acid substitutions into the MK1 *env* gene by in vitro mutagenesis based on the amino acid alignment of the MK1 and MK38 virus strains, and then compared their neutralisation resistance.

## 2. Results

### 2.1. Neutralisation of MK1 (tier 1) and #818 (tier 2) in MK1- and #818-Infected MP and HIV-1-Infected Human Pooled Plasma (HPP)

First, we sought to generalise the adaptation of HIV in humans to monkeys, because neither Ishida et al. nor Matsuda et al. examined the neutralisation resistance of SHIV-MK1 and SHIV#818 against SHIV-infected monkey plasma (MP) [5,6]. Therefore, we assessed whether MP represented HPP by comparing the neutralisation resistance of SHIV-MK1 (tier 1B), SHIV-MK38#818 (tier 2), and the control strains HIV-1 TRO (tier 2) and HIV-1 SF162 (tier 1A) with the TZM-bl assay.

**Figure 2:**
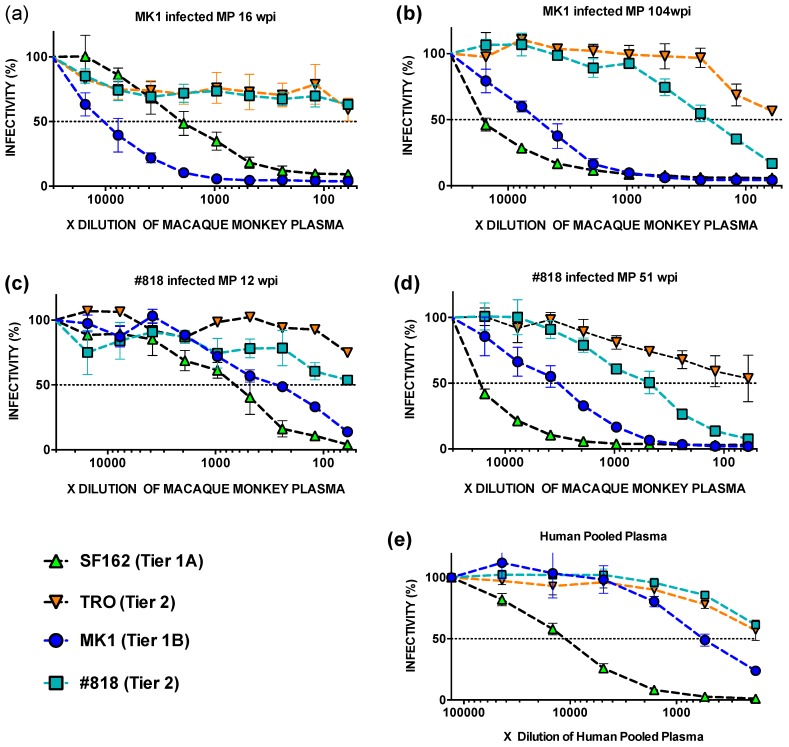
Analysis of the neutralisation resistance of each virus. (**a**–**d**) Neutralisation resistance of each virus to MM482 16 wpi, MM482 104 wpi, MM597 12 wpi, and MM597 51 wpi plasmas. After pre-incubating 100 TCID_50_ of each virus and all MP samples, TZM-bl cells were cultured with the mixture at 37 °C for 48 h and their luciferase activity was measured. All MP samples were diluted two-fold from 1:60 to 1:30,720. The values in parentheses are ID_50_. (**e**) Neutralisation resistance of each virus to pooled plasma of HIV-1-infected individuals. After pre-incubating 100 TCID_50_ of each virus and pooled plasma, TZM-bl cells were cultured with the mixture at 37 °C for 48 h and their luciferase activity was measured. Human pooled plasma was diluted three-fold from 1:180 to 1:13,1220. The values in parentheses are ID_50_.

We used tier 1 SHIV-infected MP, such as MK1 (MK1 inf. MP), to determine whether plasma induces tier 1 antibodies. While MK1 inf. MP prepared 16 weeks post-infection (wpi) efficiently neutralised MK1 (tier 1B) and SF162 (tier 1A), this plasma did not neutralise either #818 or TRO. This indicates that MK1 inf. MP 16 wpi contained tier 1, but not tier 2, antibodies (Figure 2a).

The MK1 inf. MP at 104 wpi efficiently neutralised MK1 and SF162, and contained a low titre of antibodies that neutralise #818; however, it did not neutralise TRO. Therefore, 104 wpi plasma can strongly induce tier 1A and 1B antibodies, and can trigger the induction of tier 2 antibodies for #818 (Figure 2b). MK1 inf. MP at 104 wpi could not induce tier 2 antibodies for #TRO, but it could induce tier 2 antibodies for #818 because #818 is molecular clone of MK1.

Next, we used tier 2 SHIV-infected MP (i.e., #818 inf. MP) to examine whether the macaque plasma induced tier 2 antibodies. While the antibodies elicited in #818 inf. MP at 12 weeks post #818 infection neutralised MK1 and SF162 efficiently, they did not neutralise either #818 or TRO. This indicates that #818-infected monkey 12 wpi plasma can induce tier 1A and 1B antibodies (Figure 2c). MK1 inf. MP at 16 wpi and #818 inf. MP at 16 wpi are similar.

The #818 inf. MP at 51 weeks post-#818 infection efficiently neutralised MK1(3400×) and SF162 (17,000×), and neutralised #818 to some degree (350×), but it could not neutralise TRO. This indicates that #818 inf. MP at 51 wpi contained tier 1 and 2 antibodies that neutralise #818 (Figure 2d). While #818 inf. MP at 51 wpi could induce tier 2 antibodies for #818, it could not induce tier 2 antibodies for #TRO.

We also determined the ID_50_ of SHIV-MK1 and #818 against HPP, and found that the ID_50_ of HIV-1 reference clone tier 1A SF162 was 1:11,110, while that of tier 2 HIV-TRO was 1:153. The ID_50_ of SHIV-MK1 was 1:550 and that of MK38#818 was 1:138 (Figure 2e). The ID_50_ of #818 against HPP was 3.9-fold greater than that of MK1. The neutralisation resistance patterns of MK1 and #818 against #818-infected MP at 51 wpi, MK1 infected MP at 104 wpi, and HIV-1-infected HPP were similar. These results also demonstrate that monkey experiments for HIV research remain preliminary.

### 2.2. Neutralisation of MK1 and #818 by Each Monoclonal Antibody (mAb)

**Figure 3:**
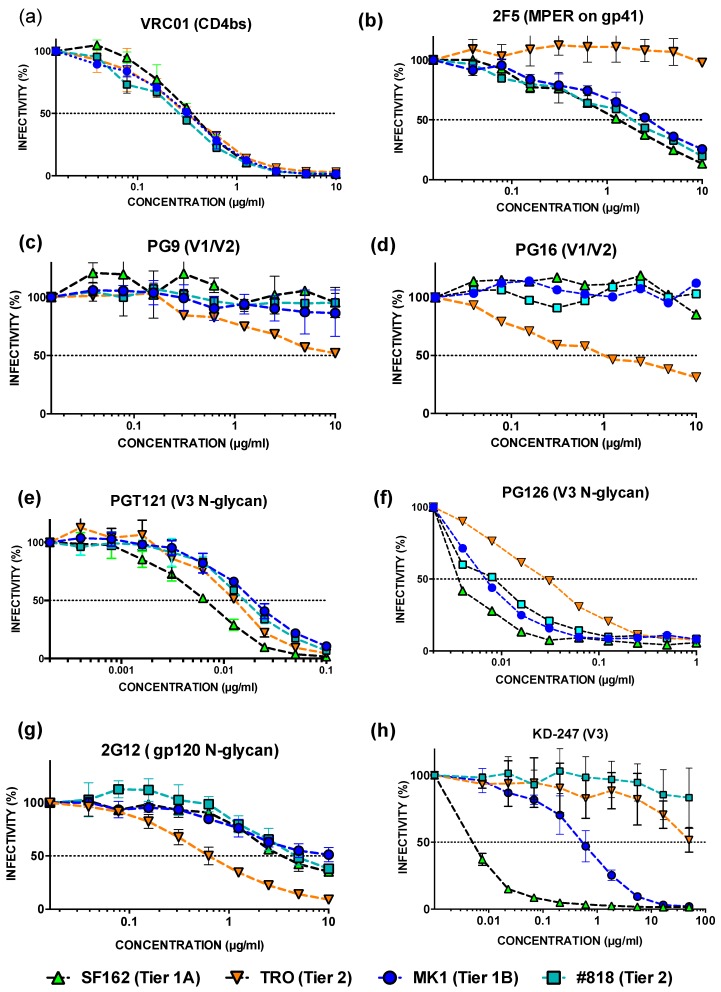
Analysis of the neutralisation resistance of each virus to eight anti-HIV-1 neutralising mAbs. After pre-incubating 100 TCID_50_ of each virus and each HIV-1-neutralising mAb, TZM-bl cells were cultured with the mixture for 48 h, after which the luciferase activity was measured. (**a**) MAb VRC01 recognizes the structure that includes CD4bs on gp120 [7]. VRC01 was diluted two-fold from 10 to 0.02 µg/mL. (**b**) 2F5 is a recombinant monoclonal antibody to HIV-1 gp41 [8]. 2F5 was diluted two-fold from 10 to 0.02 µg/mL. (**c**) PG9 is a recombinant, broadly neutralizing monoclonal antibody (bnMAb) to HIV-1 gp120, specifically to quaternary structure of V1V2 [9]. PG9 was diluted two-fold from 10 to 0.02 µg/mL. (**d**) PG16 is a recombinant, bnMAb to HIV-1 gp120, specifically to the quaternary structure of V2 [9]. PG16 was diluted two-fold from 10 to 0.02 µg/mL. (**e**) PGT 121 is a recombinant, bnMAb to HIV-1 gp120, specifically to the N332-centered oligomannose patch on the V3 loop [10]. PGT121 was diluted two-fold from 0.1 to 0.0002 µg/mL. (**f**) PGT126 is a recombinant, bnMAb to HIV-1 gp120, specifically to the N332-centered oligomannose patch of the V3 loop. PGT 126 was diluted two-fold from 1 to 0.002 µg/mL [10]. (**g**) 2G12 is a recombinant mAb to HIV-1 gp120. 2g12 contact with gp120 carbohydrates at glycosylation residues in C2, C3, C4, V3 and V4, gp120 [11,12]. 2G12 was diluted two-fold from 10 to 0.02 µg/mL. (**h**) KD-247 binds PGR epitope on V3 region and efficiently neutralizes CXCR4- and CCR5-tropic primary HIV-1 clade B including tier 2 JR-CSF virus [13]. KD247 was diluted three-fold from 50 to 0.001 µg/mL.

To characterise the neutralisation properties of MK and #818, we examined the effects of several mAbs on known epitopes to determine the dominant mAb representing HIV-1-infected HPP:

- The 50% inhibition concentrations (IC_50_) of TRO, #818, MK1, and SF162 against VRC01 (gp120 CD4bs) were within 0.3 µg mL^−1^ (Figure 3a).
- The IC_50_ of #818, MK, and SF162 against anti-HIV-1 gp41 mAb 2F5 was <3 µg mL^−1^, while the IC_50_ of TRO was > 10 µg mL^−1^ (Figure 3b). The neutralisation resistance to 2F5 is similar between MK and #818.
- The IC_50_ values of MK1 and #818 against mAb PG9 (gp120 V2 N-glycan) and mAb PG16 (gp120 V2 N-glycan) are > 10 µg mL^−1^ and are similar to each other (Figure 3c,d).
- The IC_50_ values of MK1, #818, SF162, and TRO against mAb PGT121 and PGT 126 (gp120 V3 N-glycan) were <0.1 µg mL and were similar to each other (Figure 3e,f).
- The IC_50_ values of MK1, #818, SF162, and TRO against mAb 2G12 (gp120 C2V3C3V4C4 N-glycan) were 10, 5, 3.4, and 0.6 µg/µL, respectively (Figure 3g). The IC_50_ values of MK and #818 against 2G12 were not like those against HPP.
- While the IC_50_ of #818 against KD247 mAb (IGPGR epitope on gp120 V3) is >50 µg mL^−1^, that of TRO is around 50 µg mL^−1^, the IC_50_ values of MK1 and SF162 are 0.55 and 0.047 µg mL^−1^, respectively. #818 had more than 100 times greater neutralisation resistance to KD247 than did MK1 (Figure 3h).

We found that the neutralisation analysis patterns of MK1 inf MP at 104 wpi, #818 inf. MP at 51 wpi, HIV-1 inf HPP, and KD247 were similar, while most of the Abs in MK1 inf MP at 104 wpi, #818 inf. MP at 51 wpi, and HIV-1 inf HPP were similar to KD247 mAb (Figure 2; Figure 3h). The other antibodies did not yield similar results (Figure 3). We selected the antibody closest to KD247 mAb as representative of HIV-1-infected HPP and SHIV-infected MP.

### 2.3. Neutralisation of Consensus Pseudoviruses in KD247

We analysed the sequence variation of the MK38 strain and identified four majority consensus mutations in the 13 substitutions in the #818 clone: K187E, S190N, A389T, and T437A. Then, *env* consensus mutants for MK1 were generated using a mutagenesis method: MK1+K187E (E), MK1+K187E+S190N (EN), MK1+K187E+S190N+A389T (ENT), and MK1+K187E+S190N+A389T+ T437A (ENTA). We performed neutralisation assays using KD247 mAb.

**Figure 4:**
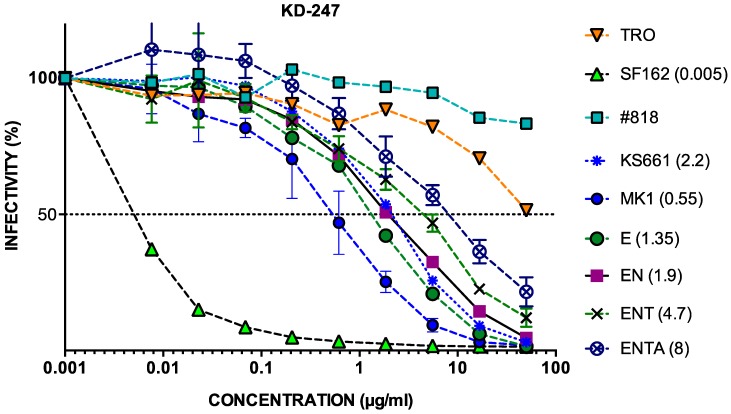
Analysis of the neutralisation resistance of each virus to anti-HIV-1 V3 KD247 Ab. After pre-incubating 100 TCID_50_ of each virus and KD247 mAb, TZM-bl cells were cultured with the mixture for 48 h, after which the luciferase activity was measured. KD247 was diluted three-fold from 50 to 0.001 µg/mL. The values in parentheses are ID_50_. The different viruses are as follows: E (MK1env K187E), EN (MK1env K187E, S190N), ENT (MK1env+K187E, S190N, A389T), and ENTA (MK1env K187E, S190N, A389T, T437A).

The IC_50_ values of MK-1 and its consensus clones against KD247 were <10 µg µL^−1^; the IC_50_ values of #818 were >50 µg µL^−1^. Like MK1, the consensus MK1s mutant clones were sensitive to KD247 mAb (Figure 4).

### 2.4. Neutralisation of MK1 Mutant Molecular Clones with 169D in KD247

None of the consensus MK1 mutant clones showed resistance similar to that of #818 against KD247. ENTA, with the four consensus MK1 mutants, had better neutralisation resistance to KD247 than did MK1 (Figure 4). The consensus MK1+ENTA has 187E, which results in a negative charge, and 190N in the V2 region of gp120 and 389T in V4, which create sites for N-glycan.

However, Matsuda et al. reported that the amino acid substitutions in the V2 region in the 14 MK38 clones were N169D, K187E, and S190N [6]. We also examined the mutations in #818 env. N169D in the V1V2 region also caused an added negative charge, like K187E. Therefore, we added N169D to the consensus MK1+ENTA using a mutagenesis method.

**Figure 5:**
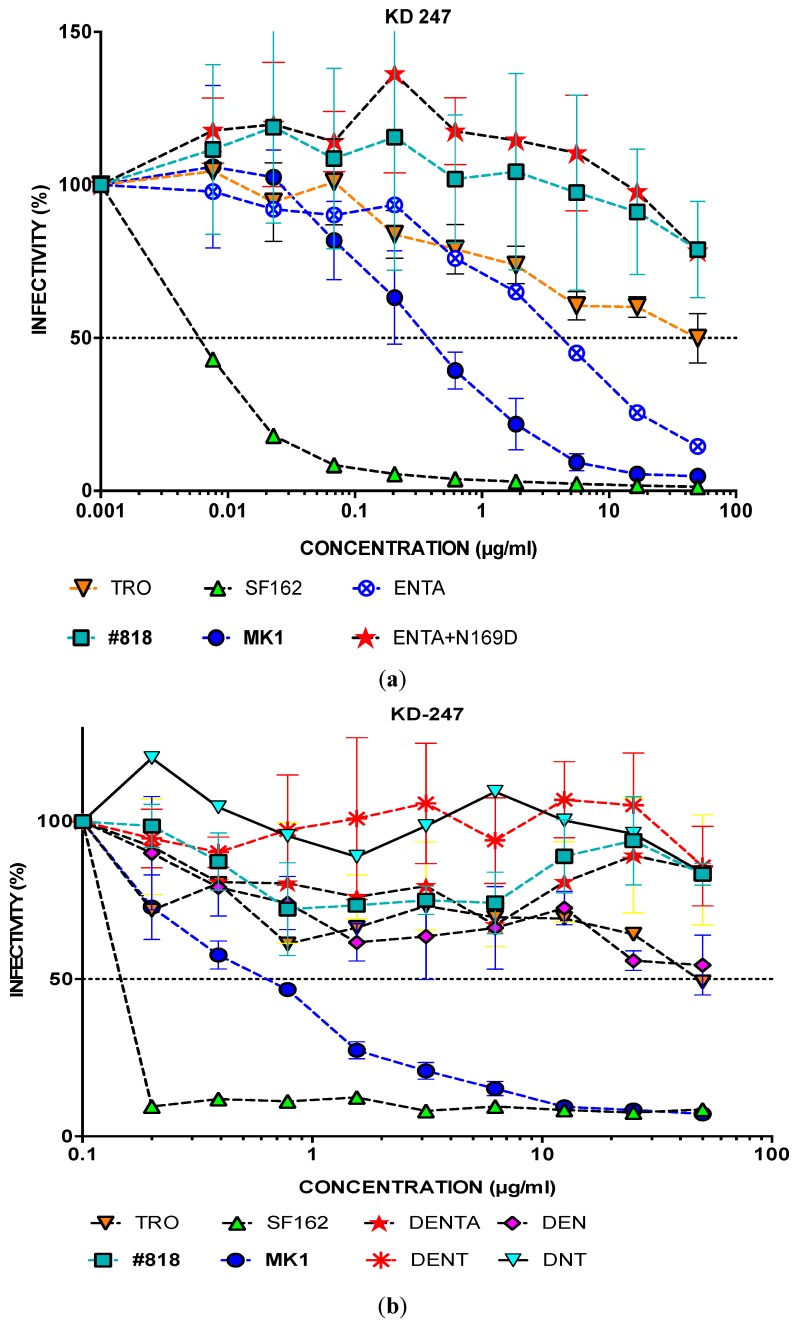

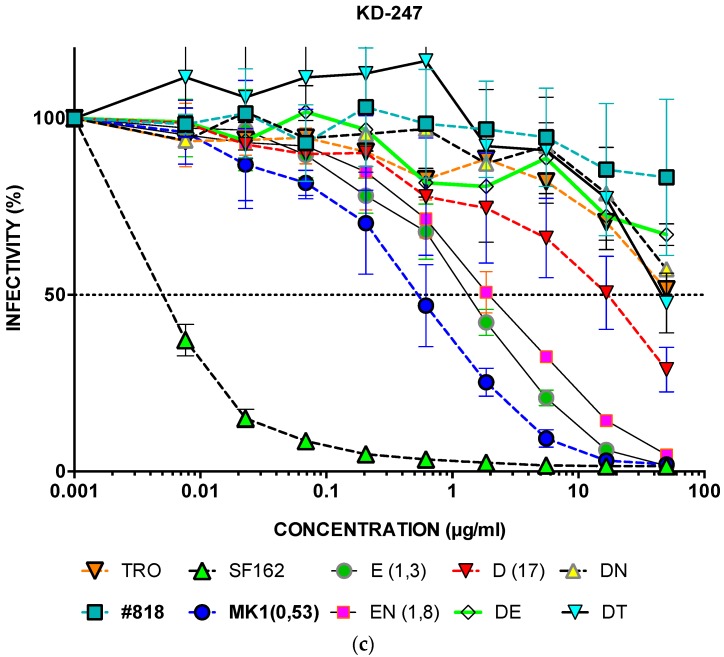
Analysis of the neutralisation resistance of each virus to anti-HIV-1 V3 KD247 mAb. After pre-incubating 100 TCID_50_ of each virus with KD247 mAb, TZM-bl cells were cultured with the mixture for 48 h, after which the luciferase activity was measured. KD247 was diluted (**a**) three-fold from 50 to 0.01 µg/mL; (**b**) two-fold from 50 to 0.1 µg/mL; or (**c**) three-fold from 50 to 0.01 µg/mL. The different viruses are as follows: ENTA (MK1env K187E, S190N, A389T, T437A), DENTA (MK1env K187E, S190N, A389T, T437A+N169D), DENT (MK1env+K187E, S190N, A389T+N169D), DNT (MK1env+N169D, S190N, A389T), MK1+DEN (MK1env+N169D, K187E, S190N), DE (MK1env+N169D, K187E), DN (MK1env+N169D, S190N), DT (MK1env+N169D, A389T), DT (MK1env+N169D, A389T), D (MK1env+N169D), and E (MK1env+K187E).

Notably, the IC_50_ of the consensus MK1+ENTA with N169D was >50 µg mL^−1^, similar to that of #818 against KD247. The IC_50_ of the consensus MK1+ENTA was <10 µg mL^−1^ (Figure 5a). The addition of 169D to MK1+ENTA increased the neutralisation resistance markedly (Figure 5a). We also added N169D to the other consensus MK1s (i.e., MK1+E, MK1+EN, MK1+ENT, and MK1+ENTA), including in various different combinations to obtain resistant MK1 with minimal mutations, including 169D. Therefore, we generated DENT (MK1env+N169D+K187E+S190N+A389T), DEN, DNT, DE, DN, DT, and D to determine which mutations are important for neutralisation resistance against KD247 when combined with N169D. We also tested neutralisation resistance against KD247 with other substitutions, i.e., N133S, S147G, N169D, N237T, D282N, S294F, T437A, and T463P with MK1, but did not see any difference from the consensus MK1s (<10 µg mL^−1^; data not shown).

The IC_50_ values of #818, DENTA, DENT, DEN, DNT, DE, DN, and DT against KD247 were similar, and were all >50 µg mL^−1^ (Figure 5b,c). While the IC_50_ of MK1 was 0.5 µg mL^−1^, that of MK1+ N169D was 17 µg mL^−1^. The IC_50_ of MK1+169D indicated more than 34 times greater neutralisation resistance to KD247 compared to MK1 (Figure 5c).

The DEN, DE, DN, and DT viruses had MK1 neutralisation resistance close to that of #818, which was similar to tier-2 TRO. Moreover, the DENTA, DENT, and DNT viruses were as MK1 neutralisation-resistant as #818 (Figure 5a–c). In summary, the consensus mutant MK1s with 169D were neutralisation-resistant against KD247. Therefore, N169D is a key substitution for acquiring neutralisation resistance.

### 2.5. Neutralisation of MK1 Mutant Molecular Clones with 169D in HPP

We already showed that most of the Abs in HIV-1-infected HPP are like KD247 mAb. The IC_50_ values of #818, DENTA, DENT, DEN, DNT, DE, DN, and DT against KD247 were >50 µg mL^−1^. Accordingly, consensus mutant MK1s with 169D might be tier 2 viruses, such as #818 and TRO. To confirm this, we also determined the ID_50_ of consensus mutant MK1s with 169D against HPP.

**Figure 6:**
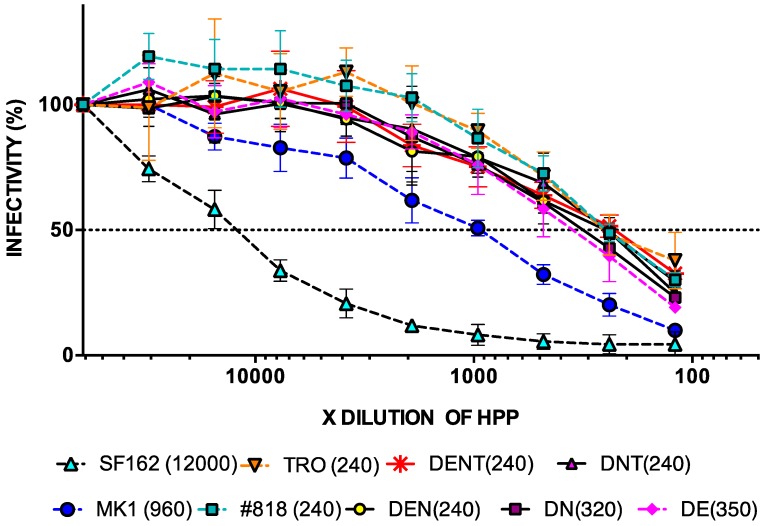
Neutralisation resistance of each virus to pooled plasma from HIV-1-infected individuals. After pre-incubating 100 TCID_50_ of each virus and pooled plasma, TZM-bl cells were cultured with the mixture at 37 °C for 48 h and their luciferase activity was measured. HPP was diluted two-fold, from 1:120 to 1:61,440. The values in parentheses are ID_50_. The different viruses are as follows: DENT (MK1env+N169D, K187E, S190N, A389T), DNT (MK1env+N169D, S190N, A389T), DEN (MK1env+N169D, K187E, S190N), DE (MK1env+N169D, K187E), and DN (MK1env+N169D, S190N).

The ID_50_ values of DENT, DEN, #818, DNT, and TRO were 1:240 against HPP, while the ID_50_ of MK1 was 1:960, and those of DN and DE were 1:320 and 1:350, respectively (Figure 6).

We discovered that DENTA, DENT, DEN, and DNT had tier 2 resistance, like #818 and TRO. We created new tier 2-resistant viruses by adding to the negative charge of 169D in MK1 with a minimum of two consensus mutations.

### 2.6. Neutralisation of MK1 Mutant Molecular Clones with 169D in SHIV-infected MP

After determining that SHIV-infected late wpi MP mimicked HIV-1-infected HPP, we also evaluated whether this was effective for molecular clones with 169D.

**Figure 7:**
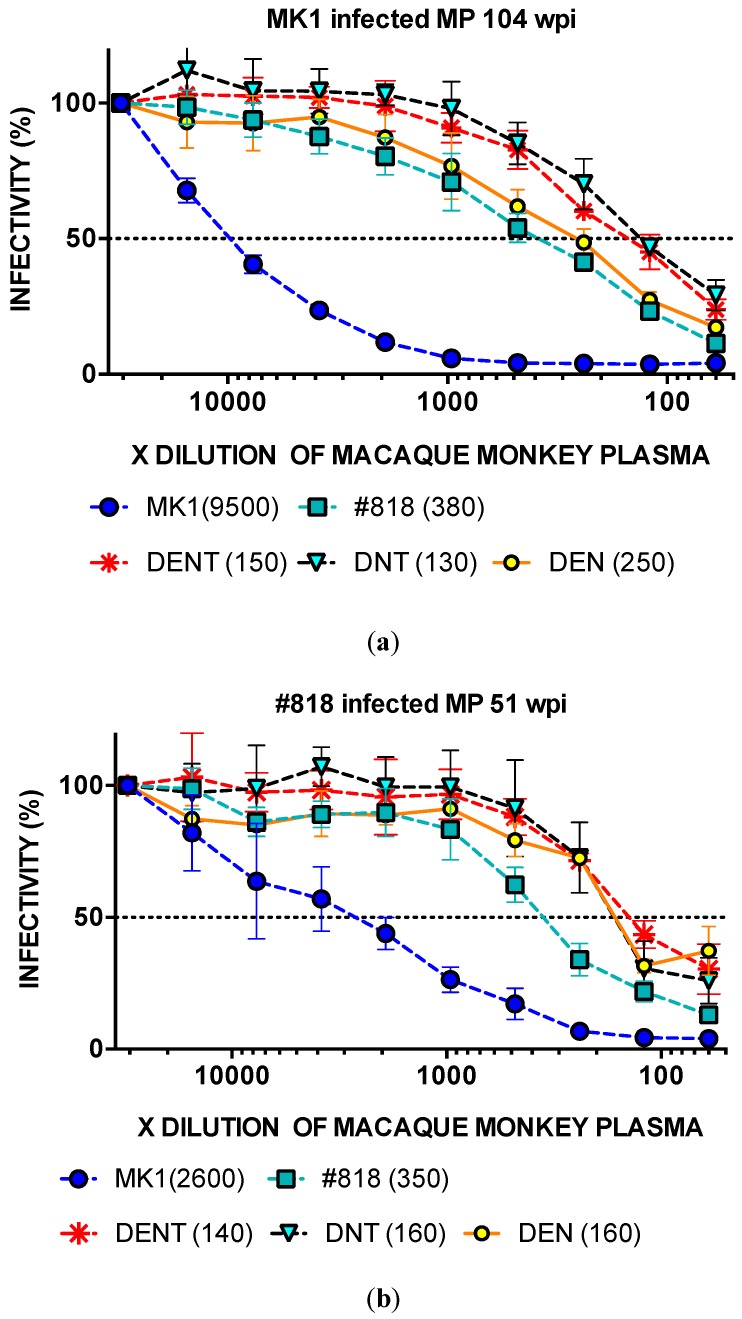
Neutralisation resistance of each virus to MM482 104 wpi and MM597 51 wpi plasma (**a**,**b**). After pre-incubating 100 TCID_50_ of each virus and all MP samples, TZM-bl cells were cultured with the mixture at 37 °C for 48 h and their luciferase activity was measured. All MP samples were diluted two-fold, from 1:60 to 1:30720. The values in parentheses are ID_50_. The different viruses are as follows: DENT (MK1env+N169D, K187E, S190N, A389T), DNT (MK1env+N169D, S190N, A389T), and DEN (MK1env+N169D, K187E, S190N).

We determined the ID_50_ values of DENT, DEN, and DNT, and compared MK1 and 818. The ID_50_ values of DENT, DNT, DEN, 818, and MK1 against MK1-infected MP at 104 wpi were 1:150, 1:130, 1:250, 1:380, and 1:9500, respectively (Figure 7a). The neutralisation resistances of all consensus MK1s with 169D were slightly higher than that for 818 against MK1 inf MP at 104 wpi.

We also determined the ID_50_ values of DENT, DNT, DEN, 818, and MK1 against #818 inf. The MP at 51 wpi for MP was 1:140, 1:160, 1:160, 1:350, and 1:2600, respectively (Figure 7b). The neutralisation resistance of all consensus MK1s with 169D was also slightly higher than that for 818 against #818 inf. MP 51 wpi (Figure 7a,b). Thus, DENT, DNT, and DEN have tier 2 resistance, like #818 and TRO.

## 3. Discussion

We investigated the cause of the resistance of #818 in detail, specifically the amino acid substitutions that make MK1 resistant. The V1V2 region of HIV strains shows great variety because it contains many substitutions, deletions, and insertions. This variety enables escape from the immune response and high resistance to specific neutralising antibodies. The other features of V1V2 are that it is somewhat longer, and has more glycosylated sites, than historical viruses [14].

The neutralisation resistance of MK1+DENT, MK1+DEN, and MK1+DNT with the V1V2 and V4 substitutions was similar to that of #818 and TRO against KD247. The negative charges at 169D and 187E, and the N-glycan 190N and 389T substitutions, are important for the neutralisation resistance. We discuss each of these substitutions in the following sections.

### 3.1. Position 169 (167 in HXB2)

While amino acid substitutions in the V1 region had no effect on neutralisation resistance, the amino acid substitutions in the V2 region did affect the neutralisation resistance. The main reason for this is that the amino acid substitutions in the V1 region obtained in our experiments did not lead to any charge changes or N-linked glycosylation sites. In comparison, the substitutions in the V2 region resulted in changes in two electrical charges (N169D and K187E) and one N-linked glycosylation site (S190N) (Figure 1). The V1/V2 fold, also known as the ‘Greek key’, contains four anti-parallel protein strands (A–D) between strands β2 and β3 of the core gp120 [15]. Arginine (R) and lysine (K) frequently form salt bridges by pairing with negatively charged aspartate (D) or glutamate (E), to create hydrogen bonds that stabilize the protein structure (salt bridges are generally K-D or D-R) [16]. We hypothesised that 169D (167 in HXB2) is important for maintaining the C strand of the Greek key because it is located at the tip of the C strand and could be involved in hydrogen bonding/salt bridges. In addition, 169D is included in peptides 167–178 of the C strands (IRDKVKKEYALF) of both TRO and #818. The formation of these hydrogen bonds/salt bridges could alter the three-dimensional conformation of the V1/V2 region. Therefore, N169D is a key substitution for acquiring neutralisation resistance.

### 3.2. Position 187 (184 HXB2)

The neutralisation resistances of #818, MK1+DENT, MK1+DEN, and MK1+DNT against KD247 were similar, and were all >50 µg/mL (Figure 5b). As shown in the figure, 187E seemed to have no effect on the neutralisation resistance. To verify this, we also performed a neutralisation resistance assay against KD247 with a minimum of two amino acid changes, including 169D (Figure 5c). The neutralisation resistances of DE, DN, DT, TRO, and #818 were all > 50 µg/mL. The neutralisation resistance of DE was closer to that of #818 compared to DN and DT. Therefore, 187E also had a positive effect on the neutralisation resistance. Like 169D, 187E can also create salt bridges/hydrogen bonds with R or K in peptides 167–178 (IRDKVKKEYALF), thus increasing the stability of the V1/V2 region.

### 3.3. Position 190 (187 HXB2)

The 190N-gylcan (187 in HXB2) in #818 increased the neutralisation resistance in combination with 169D. This is consistent with Li et al. [17], who showed that the removal of a single N-glycan at amino acid position 187 increases the sensitivity of HIV 89.6 to broadly neutralising anti-CD4bs mAb b12 and anti-V3 mAb 447-52D in vitro. Wang et al. [18] also reported that the removal of specific N-glycosylation sites has a significant effect on viral infectivity and neutralisation resistance.

### 3.4. Position 389 (388 HXB2) NXT

The addition of a glycan at position 387 (386 HXB2), with the substitution of T at 389 in #818, increased the neutralisation resistance in combination with 169D. The asparagine (N) at position 386 in HXB2 gp120, which corresponds to the N at position 387 in MK1, is not essential for protein folding or function, but is involved in immune evasion [19]. Furthermore, the loss of an N glycan at position 386 triggers a change in the structure of the V4 loops, leading to a change in the conformation of the CD4 binding site. These changes increased the sensitivity of MVC-resistant virus to sCD4 and b12 [20]. Our results showed that 386T affects V3 antigenicity by covering the V3 site with the mutated V4 region. Therefore, the structural change in V4 may greatly affect the antigenicity of gp120. This is consistent with Li et al., who indicated that the gp120 V4–V5 regions play an important role in fusion efficiency, while the V4 mutations at positions 407D and 386N regulate the resistance to CCR5 antagonists [7,21].

The neutralisation assay results against HPP and MP confirm that MK1 is a tier 1 virus and #818 is a tier 2 virus. When we characterised the neutralisation properties of MK and #818 using mAbs such as VRC01 for CD4bs, 2F5 for gp41, 2G12 for the N-glycan sites of gp120, PG9 for V1V2, and PGT121, PGT126, and KD247 for V3 sites (Figure 3), only the KD247 mAbs produced a similar pattern between HPP and MP (Figure 3). Most of the antibodies in HPP and MP are like KD247, and bind to the V3 region of gp120 *env* (Figure 1, Figure 2 and Figure 3).

**Figure 8:**
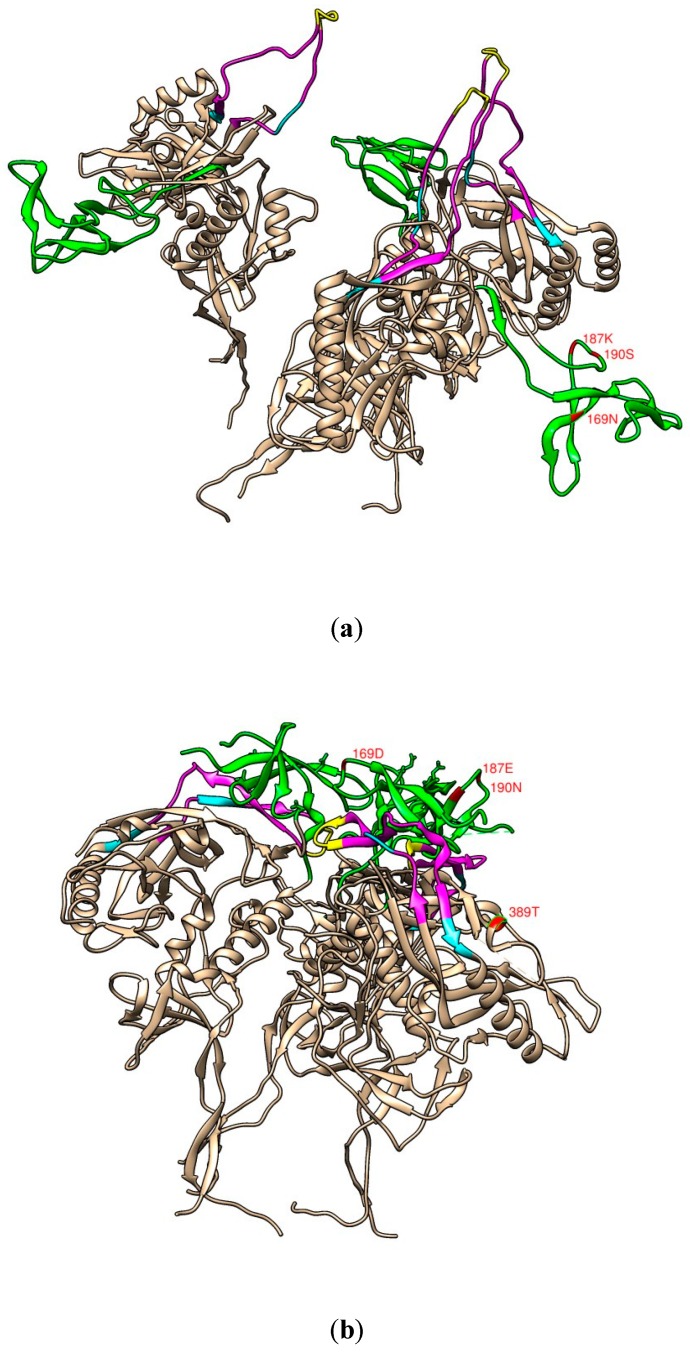
(**a**) Model of the gp120 trimeric structure of Env that mimics a tier 1 HIV (CD4 and 17b complexed with the full-length HXB2 HIV-1 Env). (**b**) Model of the gp120 trimeric structure of Env HIV (PGT145 Fab complexed with full-length AMC011 HIV-1 Env). The four most important amino acid bases associated with MK1 (**a**) and MK38#818 (**b**) are labelled in red. The models are from Protein Data Bank ID: 3J70 and 6NIJ, respectively, and were visualised using UCSF Chimera software [23,24] Both models show the V1/V2 (green) and V3 (magenta) domains and KD247 (yellow) and PGT121 (cyan) epitopes.

The mutations acquired by #818 were not located in the epitopes of the VRC01, 2F5, PGT121, or KD247 mAbs. KD247 binds the PGR epitope in the V3 region, and efficiently neutralises CXCR4- and CCR5-tropic HIV-1 in primary clade B [22]. However, while KD247 neutralised MK1 and SF162 efficiently, it did not neutralise #818 or TRO (Figure 3h). Note that the V3 site amino acid sequences of #818 and MK1 are the same, and both also have the same epitope as PGR for KD247 at the V3 site (Figure 8a).

We postulated that this problem arose because of the trimer conformation, where the neutralisation tier could be determined based on the spike in trimeric HIV-1 envelope glycoprotein. The trimer conformations of tier 2–3 HIV-1 viruses resistant to neutralising antibodies are closed and stabilised, while that of the more sensitive tier 1A strains is open, and that of the somewhat sensitive tier 1B viruses is intermediate [25,26]. Accordingly, MK1 and KS661 might have intermediate trimer structures (Figure 8b) because, as tier 1B viruses [2], they were neutralised by KD247 mAb. SF162 might also have an open trimer structure because it is a tier 1A virus. However, the KD247 mAb neutralised SF162 more than MK1 and KS661 (Figure 3h). The KD247 mAb can neutralise open and intermediate trimer structures, such as SF162, MK1, and KS661.

TRO and #818 may have closed trimer structures (Figure 8b) because they are tier 2 viruses. Neither #818 nor TRO was neutralised by the KD247 mAb. The PGR epitope of KD247 is at the apex of the V3 area, and this epitope might be under the V1V2 site (shown in yellow in Figure 8b). In 2016, Ishida et al. also reported that the neutralisation resistances against KD247 of the tier 2 viruses HIV-PVO.4 and SHIV-MK38 were >50 µg/µL. Accordingly, there is a relationship between the trimer conformation and neutralisation resistance against KD247. Although the tier 2 QH0692.42, PVO.4, AC10.0.29, REJO4541.67, RHPA4259.7, CAAN5342.A2, WITO, RHPA, TRJO, and SUMA viruses have the PGR epitope, their neutralisation resistances against KD247 were all >150 µg/µL [27].

We found that the most important amino acid substitutions and epitopes of the mAbs of MK1 and #818 fit the tier 1 (Figure 8a) and tier 2 (Figure 8b) virus models visualised with UCSF Chimera software.

Subsequently, we aimed to find amino acid substitutions that change the conformation and neutralisation resistance. Interactions between the V1V2 domains of different gp120 subunits help stabilise the trimer apex and, consequently, the entire trimer; additional interactions with the V3 domain stabilise the pre-fusion conformation of the trimer, in which the V3 domain is sequestered underneath the V1V2 loops [28]. This reduces the local V2 flexibility and improves the binding of V2-dependent bNAbs and gl-bNAbs [28]. Furthermore, overcoming *env* metastability is central to trimer-based HIV-1 vaccine design [29].

In summary, we speculate that negative charges, such as N169D (aspartic acid) and K187E, can alter the MK1 Env conformation so that the V3 region is buried. In addition, S190N+A389T can also increase the stability. As a result of substitutions N169D + K187E + S190N at V2 sites and A389T at a V4 site, the PGR epitope of KD247 at the apex of the V3 area might be under the V1V2 site (Figure 8b). A sensitive three-dimensional trimer modelling system may be needed to demonstrate our hypothesis.

## 4. Materials and Methods

### 4.1. Cell Lines

The 293T cells [30] were maintained in Dulbecco’s modified Eagle’s medium (DMEM) (Wako Pure Chemicals) supplemented with 10% fetal bovine serum (FBS) and 1 mM l-glutamine. TZM-bl cells [31] from the National Institutes of Health (NIH) AIDS Research and Reference Reagent Program were maintained in DMEM with 10% FBS, 1 mM l-glutamine, and 1 mM sodium pyruvate. Cells were harvested and passaged using trypsin/ethylenediaminetetraacetic acid solution (Nacalai Tesque, Kyoto, Japan) and were maintained at 37 °C in a humidified atmosphere containing 5% CO_2_.

### 4.2. Generation of the New Vector Plasmid “ZaferY” (pcDNA 3.1/Hygro(+) ∆KpnI∆XhoI)

To examine the size of SHIV env on agarose gel electrophoresis, we manipulated pcDNA3.1/Hygro(+), because SHIV env and the vector both contain *Kpn*I and *Xho*I sites. To eliminate the *Kpn*I site, it was digested with *Hin*dIII HF at 37 °C for 15 min. The vector was ligated with ligation mix at 16 °C for 30 min. To delete the *Xho*I site in the vector, it was digested with *Not*I and *Apa*I, blunt-ended using T4 DNA polymerase, and self-ligated. This new vector was called “ZaferY”.

### 4.3. Generation of Pesudoviruses

The pSHIV-KS661 *env* gene, pSHIV-MK1 *env* gene, and pSHIV-#818 with the pUC119 vector *env* gene were obtained from the Research Centre for Infectious Diseases Primate Laboratory of the Institute for Frontier Life and Medical Science (Kyoto University, Kyoto, Japan). A 2-kb DNA fragment containing *env* was subcloned into ZaferY vector following digestion with the restriction enzymes *Kpn*I and *Xho*I.

### 4.4. Construction of Mutant MK1 env Clones

Mutant MK1 strains were generated using the KOD-Plus-Mutagenesis Kit (Toyobo, Osaka, Japan). These included N133S, S147G (V1 region), N169D, K187E, S190N (V2 region), N237T, D282N, S294F (C2 region), A389T (V4 region), T437A (C4 region), and T463P (V5 region).

PCR consisted of an initial denaturation (94 °C for 2 min), nine amplification cycles (98 °C for 30 s, 53 °C for 30 s, and 68 °C for 8 min 24 s), and a final extension (68 °C for 8 min 24 s). The reactions consisted of 35 µL of PCR-grade water, 5 μL of KOD Plus 10× buffer, 5 µL of 2 mM dNTPs, 1.5 µL of 10 pmol/µL primers F and R, 1 µL of KOD Plus DNA Polymerase (Toyobo) and 1 µL of 50 ng/µL as template. The following primers were used for the mutagenesis assays.
A6843G (N169D)F primer (5′-GATAAGGTAAAGGAAAGAATATGCAC-3′)
R primer (5′-TCTTATGCTTGTGGTGATATAGAAA-3′)A6897G (K187E) F primer (5′-GAAAATACTAGTAATACTAAGTATAGGT-3′)
R primer (5′-TACTGGTACTACATCAAGTCTATTAAAA-3′)G6907A (S190N) F primer (5′-ATAATACTAAGTATAGGTTAATAAGTTG-3′)
R primer (5′-TAGTATTTTTTACTGGTACTACATC-3′)G7503A (A389T) F primer (5′-ACACAACTGTTTAATAGTACTTGGAATG-3′)
R primer (5′-TGTATTACAGTAGAAAAATTCCCCTCCA-3′)A6736G (N133S) F primer (5′-GTTTGAATATCACTAAGAATACTACTAATC-3′)
R primer (5′-TAGTGCAATTTAAAGTAACACAGAGTGGG-3′)A6777G (S147G) F primer (5′-GGCTGGGGAATGATGGAGGAAGGAGAAATA-3′)
R primer (5′-GCTACTAGTGAGATTAGTAGTATTCTTAGT-3′)A7048C (N237T)F primer (5′-CTGGATCAGGACCATGCACAAAT-3′)
R primer (5′-TGAATGTCTTATTGTTACACTTTAGTATCG-3′)G7182A (D282N) F primer (5′-AACAATGTTAAAACCATAATAGTACAGCTA-3′)
R primer (5′-TGTGAAATCTTCAGATCTAATTACTATGTC-3′)C7219T (S294F) F primer (5′-TTGTAGTAATTAATTGTACAAGACC-3′)
R primer (5′-ATTCATTTAGCTGTACTATTATGGT-3′)A7647G (T437A) F primer (5′-GCAGGACAAATTAGATGTTCATCAAATA-3′)
R primer (5′-GATGGGAGGGGCATACATTGCTTTTCCT-3′)

### 4.5. Isolation of MP from Infected Rhesus Macaques

In 2010, Matsuda et al. constructed R5 tropic SHIV-MK1 by introducing five amino acid substitutions in the *env* V3 area of tier 1 HIV-1 X4 tropic SHIV-KS661 based on a consensus amino acid alignment analysis of subtype B R5 HIV-1; nevertheless, MK1 was still tier 1 [6]. Therefore, they passaged MK1 intravenously in a rhesus macaque (MM482). To allow MK1 to adapt, it was passaged in vivo from macaque MM482 to one uninfected macaque (MM498) and then to a second uninfected macaque (MM504).

After these two passages, they obtained a tier 2 MK38 strain as a mixture of viruses. Three uninfected rhesus macaques (MM481, MM501, and MM502) were infected with MK38 intravenously [6]. Then, MK38#818, which was neutralisation-resistant to HIV-1-infected human plasma, like the parental SHIV-MK38, was cloned from the MK38 strain [5]. The uninfected rhesus macaque MM597 was infected by tier 2 #818 to study the pathogenesis of HIV [5]. MP was collected from the infected macaques MM481 (MK1 infected macaque) and MM597 (#818 infected macaque) and stored at −80 °C.

The Indian rhesus macaque experiments were performed in a biosafety level 3 facility in the Institute for Frontier Life and Medical Sciences Primate Laboratory, in accordance with the institutional regulations of the Committee for the Experimental Use of Non-human Primates.

### 4.6. Neutralisation Assays

To evaluate virus neutralisation resistance, we performed neutralisation assays using plasma at 16 and 104 wpi from the SHIV-MK1-infected MM482 monkey, plasma at 12 and 51 wpi from the SHIV-MK38#818-infected MM597 monkey, pooled plasma from HIV-1-infected individuals (ZeptoMetrix, Buffalo, NY, USA), and anti-HIV-1 monoclonal neutralisation antibodies. In the assays, luciferase activity was measured using TZM-bl cells [32]. The details of the neutralisation assays have been described elsewhere [5,6].

The pooled plasma of HIV-1-infected individuals was diluted three-fold, from 1:60 to 1:1180980. MK38#818- and MK1-infected MP were diluted two-fold from 1:60-1:30720. Subsequently, the ID_50_ was calculated as described earlier [25].

Six anti-HIV-1 monoclonal neutralising antibodies were used. KD247, which recognises the GPGR epitope in the V3 region of gp120, was kindly provided by the Centre for AIDS Research (Kumamoto University, Kumamoto, Japan). PGT121, 2F5, VRC01, 2G12, and PG9 were provided by Dr. Bruce Brown through the NIH AIDS Reagent Program (Germantown, MD, USA). KD247 was diluted three-fold from 50 to 0.001 µg/mL; 2F5, VRC01, 2G12, and PG9 were diluted two-fold from 10 to 0.015 µg/mL; and PGT121 was diluted two-fold from 0.1 to 0.002 µg/mL. The IC_50_ values were then calculated as described earlier [25].

## 5. Conclusions

We introduced amino acid substitutions into the envelope of MK1 by in vitro mutagenesis and then compared the neutralisation resistance to MP, HPP, and KD247 mAb with #818 in neutralisation assays using TZM-bl cells. Based on the alignment of *env* of MK1 and #818, we selected 11 substitutions: N133S, S147G (V1 region), N169D, K187E, S190N (V2 region), N237T, D282N, S294F (C2 region), A389T (V4 region), T437A (C4 region), and T463P (V5 region). The neutralisation resistance of the mutant MK1s that harboured 7 of the 11 substitutions (N133S, S147G, N237T, D282N, S294F, T437A, and T463P) did not change significantly. These substitutions did not affect any negative charges or N-glycans. However, higher neutralisation resistance was conferred by the N169D and K187E substitutions, which added negative charges, and the S190N (V2 region of gp120) and A389T (V4 region) substitutions, which created sites for N-glycan. The combinations N169D+K187E, N169D+S190N, and N169D+A389T led to MK1 having similar neutralisation resistance to #818, while the combination N169D +S190N+ A389T led to identical neutralisation resistance between MK1 and #818. The combinations without 169D were neutralisation-sensitive. Therefore, N169D of V2 region is a key substitution for acquiring neutralisation resistance against anti-V3 antibodies. These findings illuminates a genetic aspect of how HIV may evade deactivation by the B cell mediated adaptive immunity.
